# Dichlorido[1-(8-quinolylimino­meth­yl)-2-naphtholato]iron(III)

**DOI:** 10.1107/S1600536809022880

**Published:** 2009-06-20

**Authors:** Daisuke Urakami, Katsuya Inoue, Shinya Hayami

**Affiliations:** a Department of Chemistry, Graduate School of Science, Hiroshima University, Kagamiyama, Higashi–Hiroshima 739-8526, Japan; b Department of Chemistry and Institute for Advanced Materials Research, Hiroshima University, Kagamiyama, Higashi–Hiroshima 739-8526, Japan; c Department of Chemistry, Graduate School of Science and Technology, Kumamoto University, Kurokami, Kumamoto 860-8555, Japan

## Abstract

The Fe^III^ ion in the title complex, [FeCl_2_(C_20_H_13_N_2_O)], has a distorted square-pyramidal coordination formed by one O atom and two N atoms from a tridentate 1-(8-quinolylimino­meth­yl)-2-naphtholate ligand and two Cl atoms. In the crystal structure, mol­ecules form a column structure along the *a* axis through π–π stacking inter­actions, with centroid–centroid distances of 3.657 (1) and 3.818 (2) Å. Weak C—H⋯Cl inter­actions are observed between the columns.

## Related literature

For supra­molecular self-assembly, see: Crivillers & Furukawa (2009 ▶).
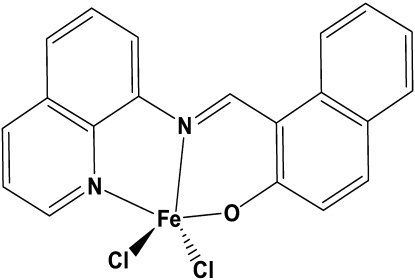


## Experimental

### 

#### Crystal data

[FeCl_2_(C_20_H_13_N_2_O)]
*M* *_r_* = 424.07
Monoclinic, 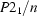

*a* = 7.6177 (5) Å
*b* = 18.5256 (11) Å
*c* = 12.2073 (7) Å
β = 91.1612 (16)°
*V* = 1722.37 (18) Å^3^
*Z* = 4
Mo *K*α radiation
μ = 1.20 mm^−1^
*T* = 293 K
0.80 × 0.20 × 0.10 mm

#### Data collection

Rigaku R-AXIS RAPID diffractometer
Absorption correction: multi-scan (**ABSCOR**; Higashi, 2001 ▶) *T*_min_ = 0.448, *T*_max_ = 0.890
17621 measured reflections
3934 independent reflections
3182 reflections with *I* > 2σ(*I*)
*R*_int_ = 0.034

#### Refinement

*R*[*F* ^2^ > 2σ(*F* ^2^)] = 0.037
*wR*(*F* ^2^) = 0.090
*S* = 1.08
3934 reflections
235 parameters
H-atom parameters constrained
Δρ_max_ = 0.44 e Å^−3^
Δρ_min_ = −0.25 e Å^−3^

### 

Data collection: *PROCESS-AUTO* (Rigaku, 1998 ▶); cell refinement: *PROCESS-AUTO*; data reduction: *CrystalClear* (Molecular Structure Corporation and Rigaku, 2002 ▶); program(s) used to solve structure: *SHELXS97* (Sheldrick, 2008 ▶); program(s) used to refine structure: *SHELXL97* (Sheldrick, 2008 ▶); molecular graphics: *Yadokari–XG* (Wakita, 2000 ▶); software used to prepare material for publication: *SHELXL97*.

## Supplementary Material

Crystal structure: contains datablocks I, global. DOI: 10.1107/S1600536809022880/is2429sup1.cif

Structure factors: contains datablocks I. DOI: 10.1107/S1600536809022880/is2429Isup2.hkl

Additional supplementary materials:  crystallographic information; 3D view; checkCIF report

## Figures and Tables

**Table 1:** Hydrogen-bond geometry (Å, °)

*D*—H⋯*A*	*D*—H	H⋯*A*	*D*⋯*A*	*D*—H⋯*A*
C14—H12⋯Cl1^i^	0.93	2.81	3.598 (2)	143
C19—H8⋯Cl1^ii^	0.93	2.86	3.656 (2)	144

Symmetry codes: (i) 
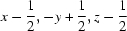
; (ii) 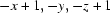.

**Fig. 1.**
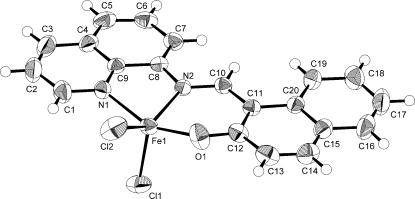
ORTEP drawing of the title complex, showing 50% probability displacement ellipsoids.

**Fig. 2.**
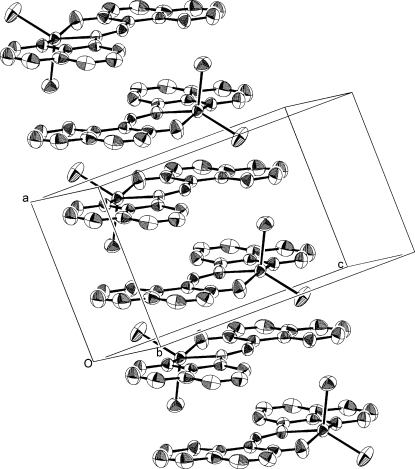
Column structure for the title complex.

**Fig. 3.**
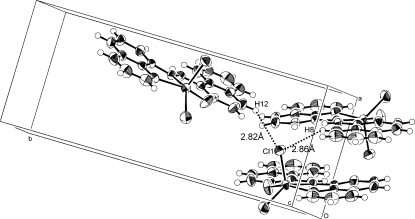
Part of the crystal structure, showing C—H···Cl interactions as dashed lines.

## supplementary crystallographic information

### Comment

Self-assembly has been recognized as a most efficient process that organizes individual molecular components into highly ordered supramolecular species (Crivillers *et al.*, 2009). The designed construction of supramolecules from molecular building blocks is noted as one of most challenging issues facing synthetic chemistry today. The method by using self-assembly is very important in developing novel molecular compounds with multi-functions. The cooperativity can be achieved by using π-π interactions as well as by using bridging ligands. We focused on a iron(III) complex with a qnal ligand [qnal = 1-(quinolin-8-yliminomethyl)-naphthalen-2-ol] having large π electron system. Here we report the synthesis and crystal structure of the title complex.

The Fe^III^ ion in the title complex, [Fe(qnal)Cl_2_], has a distorted five coordination environment formed by one O atom and two N atoms from a qnal ligand, and two Cl atoms. The Fe—O bond length is shortest and the Fe—Cl bond length is longest. The π–π contacts between the benzene and pyridine rings, *Cg*1···*Cg*3^i^ and *Cg*2···*Cg*3^ii^ [symmetry codes: (i) -*x*, -*y*, 1 - *z*; (ii) 1 - *x*, -*y*, 1 - *z*, where *Cg*1, *Cg*2, *Cg*3 are centroids of the rings (N1/C1–C4/C9), (C4–C9) and (C11–/C15/C20), respectively] may stabilize the structure, with centroid-centroid distances of 3.657 (1) and 3.818 (2) Å, respectively. The molecules form a column structure by π-π stacking along the *a* axis. Three dimensional network is formed through C—H···Cl interactions between columns.

### Experimental

The ligand molecule, qnal, was prepared from 8-aminoquinoline (4.2 mg, 0.03 mmol) and 2-hydroxy-1-naphthaldehyde (5.1 mg, 0.03 mmol), which were mixed in 10 ml methanol and heating on a oil bath for about 2 h under reflux. The title complex was prepared by slow diffusion of qnal (9.0 mg, 0.03 mmol) and FeCl_3_ (4.9 mg, 0.03 mmol) in methanol by using a H-form tube. After about one week, single crystals were obtained as black needles.

### Refinement

All H atoms were positioned geometrically (C—H = 0.93 Å) and were refined as riding, with *U*_iso_(H) = 1.2*U*_eq_(C).

### Crystal data

**Table:**

[FeCl_2_(C_20_H_13_N_2_O)]	*F*(000) = 860
*M**_r_* = 424.07	*D*_x_ = 1.635 Mg m^−^^3^
Monoclinic, *P*2_1_/*n*	Mo *K*α radiation, λ = 0.71075 Å
Hall symbol: -P 2yn	Cell parameters from 15565 reflections
*a* = 7.6177 (5) Å	θ = 3.1–27.7°
*b* = 18.5256 (11) Å	µ = 1.20 mm^−^^1^
*c* = 12.2073 (7) Å	*T* = 293 K
β = 91.1612 (16)°	Needle, black
*V* = 1722.37 (18) Å^3^	0.80 × 0.20 × 0.10 mm
*Z* = 4	

### Data collection

**Table:**

Rigaku R-AXIS RAPID diffractometer	3934 independent reflections
Radiation source: fine-focus sealed tube	3182 reflections with *I* > 2σ(*I*)
graphite	*R*_int_ = 0.034
ω scans	θ_max_ = 27.5°, θ_min_ = 3.1°
Absorption correction: multi-scan (*ABSCOR*; Higashi, 2001)	*h* = −9→9
*T*_min_ = 0.448, *T*_max_ = 0.890	*k* = −24→23
17621 measured reflections	*l* = −15→15

### Refinement

**Table:**

Refinement on *F*^2^	Primary atom site location: structure-invariant direct methods
Least-squares matrix: full	Secondary atom site location: difference Fourier map
*R*[*F*^2^ > 2σ(*F*^2^)] = 0.037	Hydrogen site location: inferred from neighbouring sites
*wR*(*F*^2^) = 0.090	H-atom parameters constrained
*S* = 1.08	*w* = 1/[σ^2^(*F*_o_^2^) + (0.0395*P*)^2^ + 0.7323*P*] where *P* = (*F*_o_^2^ + 2*F*_c_^2^)/3
3934 reflections	(Δ/σ)_max_ = 0.001
235 parameters	Δρ_max_ = 0.44 e Å^−^^3^
0 restraints	Δρ_min_ = −0.25 e Å^−^^3^

### Special details

**Table:**

Geometry. All e.s.d.'s (except the e.s.d. in the dihedral angle between two l.s. planes) are estimated using the full covariance matrix. The cell e.s.d.'s are taken into account individually in the estimation of e.s.d.'s in distances, angles and torsion angles; correlations between e.s.d.'s in cell parameters are only used when they are defined by crystal symmetry. An approximate (isotropic) treatment of cell e.s.d.'s is used for estimating e.s.d.'s involving l.s. planes.
Refinement. Refinement of *F*^2^ against ALL reflections. The weighted *R*-factor *wR* and goodness of fit *S* are based on *F*^2^, conventional *R*-factors *R* are based on *F*, with *F* set to zero for negative *F*^2^. The threshold expression of *F*^2^ > σ(*F*^2^) is used only for calculating *R*-factors(gt) *etc*. and is not relevant to the choice of reflections for refinement. *R*-factors based on *F*^2^ are statistically about twice as large as those based on *F*, and *R*- factors based on ALL data will be even larger.

### Fractional atomic coordinates and isotropic or equivalent isotropic displacement parameters (Å^2^)

**Table:**

	*x*	*y*	*z*	*U*_iso_*/*U*_eq_	
Fe1	0.16122 (4)	0.072865 (16)	0.67792 (2)	0.03433 (11)	
C1	0.1279 (3)	−0.04180 (15)	0.8607 (2)	0.0498 (6)	
H1	0.0834	−0.0042	0.9022	0.060*	
C2	0.1519 (4)	−0.10907 (17)	0.9103 (2)	0.0599 (7)	
H2	0.1216	−0.1161	0.9830	0.072*	
C3	0.2195 (4)	−0.16364 (16)	0.8515 (2)	0.0562 (7)	
H3	0.2361	−0.2086	0.8839	0.067*	
C4	0.2653 (3)	−0.15321 (13)	0.74142 (19)	0.0409 (5)	
C5	0.3412 (3)	−0.20622 (12)	0.6755 (2)	0.0485 (6)	
H4	0.3632	−0.2521	0.7035	0.058*	
C6	0.3825 (3)	−0.19030 (12)	0.5702 (2)	0.0451 (6)	
H5	0.4356	−0.2254	0.5276	0.054*	
C7	0.3470 (3)	−0.12257 (12)	0.52462 (19)	0.0401 (5)	
H6	0.3743	−0.1136	0.4520	0.048*	
C8	0.2723 (3)	−0.06922 (10)	0.58598 (17)	0.0306 (4)	
C9	0.2340 (3)	−0.08413 (11)	0.69642 (17)	0.0329 (4)	
C10	0.2480 (3)	0.01983 (11)	0.44882 (17)	0.0321 (4)	
H7	0.2835	−0.0167	0.4020	0.038*	
C11	0.2188 (3)	0.08832 (11)	0.40091 (17)	0.0327 (4)	
C12	0.1502 (3)	0.14570 (12)	0.46229 (19)	0.0399 (5)	
C13	0.1253 (3)	0.21425 (13)	0.4125 (2)	0.0495 (6)	
H13	0.0767	0.2516	0.4526	0.059*	
C14	0.1709 (3)	0.22598 (14)	0.3084 (2)	0.0524 (7)	
H12	0.1570	0.2719	0.2789	0.063*	
C15	0.2392 (3)	0.17054 (13)	0.24256 (19)	0.0441 (6)	
C16	0.2835 (4)	0.18369 (17)	0.1329 (2)	0.0600 (8)	
H11	0.2733	0.2303	0.1052	0.072*	
C17	0.3403 (4)	0.13057 (19)	0.0673 (2)	0.0655 (8)	
H10	0.3656	0.1400	−0.0055	0.079*	
C18	0.3606 (4)	0.06130 (17)	0.1099 (2)	0.0586 (7)	
H9	0.3989	0.0244	0.0647	0.070*	
C19	0.3254 (3)	0.04645 (14)	0.21726 (19)	0.0450 (6)	
H8	0.3441	0.0001	0.2444	0.054*	
C20	0.2612 (3)	0.10040 (12)	0.28673 (18)	0.0354 (5)	
N1	0.1657 (2)	−0.02918 (10)	0.75694 (14)	0.0366 (4)	
N2	0.2306 (2)	0.00218 (9)	0.55195 (14)	0.0305 (4)	
O1	0.1072 (3)	0.13872 (9)	0.56416 (14)	0.0559 (5)	
Cl1	0.40989 (8)	0.11675 (4)	0.74667 (6)	0.05373 (18)	
Cl2	−0.06535 (9)	0.11031 (4)	0.77771 (7)	0.0629 (2)	

### Atomic displacement parameters (Å^2^)

**Table:**

	*U*^11^	*U*^22^	*U*^33^	*U*^12^	*U*^13^	*U*^23^
Fe1	0.03643 (18)	0.03247 (18)	0.03430 (17)	0.00072 (13)	0.00555 (13)	−0.00741 (13)
C1	0.0496 (15)	0.0636 (17)	0.0365 (12)	−0.0030 (13)	0.0074 (11)	0.0006 (12)
C2	0.0656 (18)	0.076 (2)	0.0387 (14)	−0.0091 (16)	0.0045 (13)	0.0150 (14)
C3	0.0592 (17)	0.0552 (16)	0.0539 (15)	−0.0136 (13)	−0.0071 (13)	0.0222 (13)
C4	0.0372 (12)	0.0376 (12)	0.0476 (13)	−0.0079 (10)	−0.0082 (10)	0.0090 (10)
C5	0.0510 (15)	0.0290 (12)	0.0650 (16)	−0.0031 (11)	−0.0136 (13)	0.0054 (11)
C6	0.0474 (14)	0.0306 (11)	0.0569 (15)	0.0025 (10)	−0.0076 (12)	−0.0099 (10)
C7	0.0459 (13)	0.0345 (12)	0.0400 (12)	0.0007 (10)	0.0006 (10)	−0.0067 (9)
C8	0.0299 (10)	0.0264 (10)	0.0354 (11)	−0.0023 (8)	−0.0013 (8)	−0.0019 (8)
C9	0.0282 (10)	0.0336 (11)	0.0367 (11)	−0.0055 (9)	−0.0026 (8)	0.0006 (9)
C10	0.0328 (11)	0.0313 (11)	0.0322 (10)	0.0007 (9)	0.0034 (8)	−0.0047 (8)
C11	0.0314 (11)	0.0317 (11)	0.0348 (11)	−0.0002 (9)	−0.0015 (9)	−0.0010 (8)
C12	0.0397 (12)	0.0377 (12)	0.0420 (12)	0.0075 (10)	−0.0076 (10)	−0.0035 (10)
C13	0.0530 (15)	0.0365 (13)	0.0583 (16)	0.0138 (11)	−0.0138 (12)	−0.0061 (11)
C14	0.0528 (16)	0.0383 (13)	0.0654 (18)	0.0043 (11)	−0.0165 (13)	0.0139 (12)
C15	0.0390 (13)	0.0461 (14)	0.0466 (13)	−0.0034 (11)	−0.0099 (10)	0.0126 (11)
C16	0.0582 (17)	0.0689 (19)	0.0525 (16)	−0.0071 (15)	−0.0085 (14)	0.0291 (14)
C17	0.0636 (19)	0.092 (2)	0.0408 (14)	−0.0143 (17)	0.0042 (13)	0.0187 (15)
C18	0.0532 (16)	0.080 (2)	0.0431 (14)	−0.0083 (14)	0.0114 (12)	−0.0013 (14)
C19	0.0465 (14)	0.0495 (14)	0.0391 (12)	−0.0061 (11)	0.0077 (11)	0.0021 (11)
C20	0.0307 (11)	0.0381 (11)	0.0372 (11)	−0.0044 (9)	−0.0033 (9)	0.0049 (9)
N1	0.0371 (10)	0.0410 (10)	0.0317 (9)	−0.0036 (8)	0.0040 (8)	−0.0004 (8)
N2	0.0339 (9)	0.0276 (8)	0.0301 (9)	0.0004 (7)	0.0020 (7)	−0.0019 (7)
O1	0.0798 (13)	0.0474 (10)	0.0406 (9)	0.0282 (9)	0.0024 (9)	−0.0061 (8)
Cl1	0.0437 (3)	0.0501 (4)	0.0674 (4)	−0.0116 (3)	0.0018 (3)	−0.0121 (3)
Cl2	0.0552 (4)	0.0489 (4)	0.0858 (5)	0.0031 (3)	0.0354 (4)	−0.0098 (3)

### Geometric parameters (Å, °)

**Table:**

Fe1—O1	1.8876 (17)	C9—N1	1.367 (3)
Fe1—N2	2.0957 (17)	C10—C11	1.413 (3)
Fe1—N1	2.1223 (19)	C10—N2	1.310 (3)
Fe1—Cl1	2.2111 (7)	C10—H7	0.9300
Fe1—Cl2	2.2426 (7)	C11—C12	1.407 (3)
C1—C2	1.396 (4)	C11—C20	1.454 (3)
C1—N1	1.325 (3)	C12—C13	1.419 (3)
C1—H1	0.9300	C12—O1	1.299 (3)
C2—C3	1.349 (4)	C13—C14	1.342 (4)
C2—H2	0.9300	C13—H13	0.9300
C3—C4	1.408 (3)	C14—C15	1.410 (4)
C3—H3	0.9300	C14—H12	0.9300
C4—C5	1.402 (3)	C15—C16	1.408 (4)
C4—C9	1.411 (3)	C15—C20	1.416 (3)
C5—C6	1.362 (4)	C16—C17	1.346 (4)
C5—H4	0.9300	C16—H11	0.9300
C6—H5	0.9300	C17—H10	0.9300
C6—C7	1.397 (3)	C18—C17	1.392 (4)
C7—C8	1.371 (3)	C18—H9	0.9300
C7—H6	0.9300	C19—C18	1.371 (3)
C8—C9	1.412 (3)	C19—H8	0.9300
C8—N2	1.420 (3)	C20—C19	1.405 (3)
			
Fe1—N1—C1	126.14 (17)	C11—C20—C15	118.6 (2)
Fe1—N1—C9	114.86 (14)	C11—C20—C19	123.8 (2)
Fe1—N2—C8	115.18 (13)	C12—C11—C20	119.1 (2)
Fe1—N2—C10	125.53 (14)	C12—C13—H13	119.5
Fe1—O1—C12	135.52 (15)	C13—C12—O1	117.7 (2)
C1—C2—C3	119.1 (2)	C13—C14—C15	121.8 (2)
C1—C2—H2	120.5	C13—C14—H12	119.1
C1—N1—C9	118.5 (2)	C14—C13—C12	121.1 (2)
C2—C1—N1	122.9 (3)	C14—C13—H13	119.5
C2—C1—H1	118.5	C14—C15—C20	119.6 (2)
C2—C3—C4	120.7 (2)	C15—C14—H12	119.1
C2—C3—H3	119.7	C15—C16—H11	119.2
C3—C2—H2	120.5	C15—C20—C19	117.6 (2)
C3—C4—C5	124.4 (2)	C16—C15—C14	120.9 (2)
C3—C4—C9	116.9 (2)	C16—C15—C20	119.5 (2)
C4—C3—H3	119.7	C16—C17—C18	119.1 (3)
C4—C5—C6	119.8 (2)	C16—C17—H10	120.4
C4—C5—H4	120.1	C17—C16—C15	121.7 (3)
C4—C9—C8	120.8 (2)	C17—C16—H11	119.2
C4—C9—N1	121.9 (2)	C17—C18—C19	121.3 (3)
C5—C4—C9	118.7 (2)	C17—C18—H9	119.3
C5—C6—C7	121.6 (2)	C18—C17—H10	120.4
C5—C6—H5	119.2	C18—C19—C20	120.8 (3)
C6—C5—H4	120.1	C18—C19—H8	119.6
C6—C7—C8	120.6 (2)	C19—C18—H9	119.3
C6—C7—H6	119.7	C20—C19—H8	119.6
C7—C6—H5	119.2	N1—Fe1—N2	77.00 (7)
C7—C8—C9	118.47 (19)	N1—Fe1—O1	155.70 (8)
C7—C8—N2	127.17 (19)	N1—Fe1—Cl1	98.59 (5)
C8—C7—H6	119.7	N1—Fe1—Cl2	91.95 (5)
C8—C9—N1	117.29 (19)	N1—C1—H1	118.5
C8—N2—C10	119.16 (17)	N2—Fe1—O1	85.32 (7)
C9—C8—N2	114.34 (18)	N2—Fe1—Cl1	106.34 (5)
C10—C11—C12	121.0 (2)	N2—Fe1—Cl2	143.16 (5)
C10—C11—C20	119.91 (19)	N2—C10—H7	116.4
C11—C10—N2	127.14 (19)	O1—Fe1—Cl1	102.35 (7)
C11—C10—H7	116.4	O1—Fe1—Cl2	92.36 (6)
C11—C12—C13	119.7 (2)	Cl1—Fe1—Cl2	110.06 (3)
C11—C12—O1	122.6 (2)		

### Hydrogen-bond geometry (Å, °)

**Table:**

*D*—H···*A*	*D*—H	H···*A*	*D*···*A*	*D*—H···*A*
C14—H12···Cl1^i^	0.93	2.81	3.598 (2)	143
C19—H8···Cl1^ii^	0.93	2.86	3.656 (2)	144

Symmetry codes: (i) *x*−1/2, −*y*+1/2, *z*−1/2; (ii) −*x*+1, −*y*, −*z*+1.

## References

[bb1] Crivillers, N. & Furukawa, S. (2009). *J. Am. Chem. Soc.* **131**, 6246–6252. 10.1021/ja900453n 19361165

[bb2] Higashi, T. (2001). *ABSCOR* Rigaku Corporation, Tokyo, Japan.

[bb3] Molecular Structure Corporation and Rigaku (2002). *CrystalClear* MSC, The Woodlands, Texas, USA, and Rigaku Corporation, Tokyo, Japan.

[bb4] Rigaku (1998). *PROCESS-AUTO* Rigaku Corporation, Tokyo, Japan.

[bb5] Sheldrick, G. M. (2008). *Acta Cryst* A**64**, 112–122. 10.1107/S0108767307043930 18156677

[bb6] Wakita, K. (2000). *Yadokari–XG* Department of Chemistry, Graduate School of Science, The University of Tokyo, Japan.

